# Central Nervous System Compartmentalization of HIV-1 Subtype C Variants Early and Late in Infection in Young Children

**DOI:** 10.1371/journal.ppat.1003094

**Published:** 2012-12-27

**Authors:** Christa Buckheit Sturdevant, Anna Dow, Cassandra B. Jabara, Sarah B. Joseph, Gretja Schnell, Nobutoki Takamune, Macpherson Mallewa, Robert S. Heyderman, Annelies Van Rie, Ronald Swanstrom

**Affiliations:** 1 Department of Microbiology and Immunology, University of North Carolina at Chapel Hill, Chapel Hill, North Carolina, United States of America; 2 Department of Epidemiology, University of North Carolina at Chapel Hill, Gillings School of Global Public Health, Chapel Hill, North Carolina, United States of America; 3 Department of Biology, University of North Carolina at Chapel Hill, Chapel Hill, North Carolina, United States of America; 4 UNC Center for AIDS Research, University of North Carolina at Chapel Hill, Chapel Hill, North Carolina, United States of America; 5 Lineberger Comprehensive Cancer Center, University of North Carolina at Chapel Hill, Chapel Hill, North Carolina, United States of America; 6 Department of Immunology, University of Washington, Seattle, Washington, United States of America; 7 Department of Pharmaceutical Biochemistry, Kumamoto University, Kumamoto, Japan; 8 Malawi-Liverpool-Wellcome Trust Clinical Research Program, University of Malawi College of Medicine, Blantyre, Malawi; 9 Department of Biochemistry and Biophysics, University of North Carolina at Chapel Hill, Chapel Hill, North Carolina, United States of America; NIH/NIAID, United States of America

## Abstract

HIV-1 subtype B replication in the CNS can occur in CD4+ T cells or macrophages/microglia in adults. However, little is known about CNS infection in children or the ability of subtype C HIV-1 to evolve macrophage-tropic variants. In this study, we examined HIV-1 variants in ART-naïve children aged three years or younger to determine viral genotypes and phenotypes associated with HIV-1 subtype C pediatric CNS infection. We examined HIV-1 subtype C populations in blood and CSF of 43 Malawian children with neurodevelopmental delay or acute neurological symptoms. Using single genome amplification (SGA) and phylogenetic analysis of the full-length *env* gene, we defined four states: equilibrated virus in blood and CSF (n = 20, 47%), intermediate compartmentalization (n = 11, 25%), and two distinct types of compartmentalized CSF virus (n = 12, 28%). Older age and a higher CSF/blood viral load ratio were associated with compartmentalization, consistent with independent replication in the CNS. Cell tropism was assessed using pseudotyped reporter viruses to enter a cell line on which CD4 and CCR5 receptor expression can be differentially induced. In a subset of compartmentalized cases (n = 2, 17%), the CNS virus was able to infect cells with low CD4 surface expression, a hallmark of macrophage-tropic viruses, and intermediate compartmentalization early was associated with an intermediate CD4 entry phenotype. Transmission of multiple variants was observed for 5 children; in several cases, one variant was sequestered within the CNS, consistent with early stochastic colonization of the CNS by virus. Thus we hypothesize two pathways to compartmentalization: early stochastic sequestration in the CNS of one of multiple variants transmitted from mother to child, and emergence of compartmentalized variants later in infection, on average at age 13.5 months, and becoming fully apparent in the CSF by age 18 months. Overall, compartmentalized viral replication in the CNS occurred in half of children by year three.

## Author Summary

Genetically compartmentalized human immunodeficiency virus type 1 (HIV-1) subtype B populations can be variably detected in the cerebrospinal fluid (CSF) of adults. Compartmentalization is indicative of local CNS replication, and late in disease is linked to HIV-associated dementia (HAD). Compartmentalized viral populations can comprise either CCR5-using T cell-tropic or macrophage-tropic virus. Little is known about CNS infection in children or the ability of subtype C HIV-1 to evolve macrophage-tropic variants. We examined viral populations in the blood and CSF of HIV-1 subtype C-infected children. We found an intermediate level of compartmentalization in about half of the children under 18 months of age. About 50% of children older than 18 months had clearly compartmentalized virus in the CSF/CNS, and in some cases CSF virus evolved a low CD4 entry phenotype. In some of the children two variants were transmitted from the mother. In several of these cases one of the transmitted viruses was replicating in the CNS while the other was found predominantly in the blood/periphery. Our results suggest that compartmentalized CSF/CNS populations can be detected in up to 50% of children by year three, either established early in the infection or through sequestration of a transmitted variant within the CNS.

## Introduction

Human immunodeficiency virus type 1 (HIV-1) infection of the central nervous system (CNS) can occur shortly after transmission, and compartmentalized HIV-1 variants, genetically distinct from the virus in the blood, can be detected in the cerebrospinal fluid (CSF) in some individuals throughout the course of infection. Detection of compartmentalized CSF viral populations during primary infection suggests that compartmentalization can occur early in the absence of overt neurological symptoms [1]. Extensive compartmentalization of HIV-1 has been shown to be a strong indicator of HIV-1 neuropathogenesis contributing to HIV-1-associated dementia (HAD) [2], [3], [4], [5], while intermediate levels of compartmentalization are associated with either an asymptomatic state or less severe forms of HIV-1-associated neurological disease [1], [3]. Although a longitudinal link has not been made, these results raise the possibility that early detection of compartmentalized CSF variants may identify subjects with a higher risk of developing HIV-1-associated neurological complications.

Compartmentalized HIV-1 subtype B CNS populations can be either CCR5 (R5)-using T cell-tropic or macrophage-tropic [6], [7], [8], [9], [10]. Macrophage-tropic HIV-1 variants are characterized by the ability to infect cells with low CD4 surface expression [11], [12], [13], are poorly represented in the blood [9], are not transmitted [14], [15], and decay slowly following initiation of highly active antiretroviral therapy (HAART) [16], [17], unlike the rapid decay of virus replicating in activated CD4+ T cells within the blood [16], [18], [19]. While extensive research has been conducted on HIV-1 subtype B CNS infections, little is known about CNS compartmentalization of HIV-1 subtype C (the most common subtype worldwide) or the ability of HIV-1 subtype C to evolve to use low levels of CD4 for entry. Previous studies have reported significant differences in the subtype B and C envelope glycoproteins and suggested that subtype C may be less neuropathogenic than subtype B [20], [21], [22]. Additional studies on HIV-1 subtype C CNS infections are needed to improve our knowledge on HIV-1 subtype C pathogenesis, CNS compartmentalization and entry tropism.

Information on the genetic and phenotypic characteristics of HIV-1 within the CNS of infants and young children is scarce. HIV-1 CNS disease is often an AIDS-defining illness in children [23], [24], [25], and this implies that early infection of the CNS may be important in the pathogenesis of HIV-1 infection in infants [25]. Understanding the dynamics of pediatric HIV-1 CNS replication is thus of critical importance. We examined HIV-1 variants in 43 ART-naïve Malawian children aged three years or younger to determine the viral genotypes and phenotypes associated with HIV-1 subtype C pediatric CNS infection. We observed intermediate, minor compartmentalization in 25% and distinct, compartmentalized CSF variants in 28% of children. Older age and a higher CSF/blood viral load ratio were associated with CNS compartmentalization, with genetic evidence suggesting outgrowth of the compartmentalized variant starting at around 13 months of age. Transmission of multiple variants had occurred in 5 children, of which 4 had one variant sequestered within the CNS, consistent with early stochastic colonization of the CNS by the virus. Finally, we showed that genetically compartmentalized R5 virus with the ability to infect cells with low CD4 surface expression, a hallmark of macrophage-tropic viruses, can evolve in HIV-1 subtype C CNS infection in young children, although this was not common in the first three years of life. Overall we found that by 3 years of age, 50% of children infected with HIV-1 subtype C had virus independently replicating in the CNS.

## Results

### Study design

We examined viral populations in paired peripheral blood and CSF samples collected from 43 HIV-infected Malawian children presenting with either neurodevelopmental delay or acute neurologic symptoms (neurodevelopmental delay will be examined in a separate study). Subjects were infected with HIV-1 subtype C, were ART naïve, and ranged in age from 3 to 35 months. Blood viral loads ranged from 4,344 to >80,000,000 copies/mL and CSF viral loads ranged from 56 to 4,745,000 copies/mL. The virological characteristics for each subject are summarized in Table 1. Infection is assumed to be by a vertical route but the fractions infected prepartum, intrapartum, or postpartum are not known. To asses HIV-1 genetic compartmentalization, cDNA templates were generated from extracted blood and CSF viral RNA and used in single genome amplification (SGA) of the full-length viral *env* gene [26], [27], [28], [29], [30]. A mean of 19 amplicons were analyzed per sample. The sequence of the entire *env* gene was determined for each amplicon and phylogenetic analysis was completed.

**Table 1:** Subject population virological and phylogenetic characteristics.

Subject ID	Age (mo)	VL Plasma^a^	VL CSF^b^	VL ratio^c^	% Comp^d^	*P* Value^e^	CSF compart^h^	TMRCA (mo)^i^	Number transm^k^	Comp TMRCA (mo)^l^
3006	35	5.92	3.01	0.001	0	0.158	Eq	14	1	N/A
3032	4	6.79	4.64	0.007	0	0.379	Eq	3	1	N/A
3036	6	6.36	3.42	0.001	0	0.168	Eq	7	1	N/A
3040	15	5.47	3.84	0.024	0	0.023^f^	Eq	4	1	N/A
4001	22	5.48	3.22	0.006	0	0.458	Eq	18	1	N/A
4014	15	7.01	5.08	0.012	0	0.349	Eq	12	1	N/A
4015	22	6.19	4.25	0.011	0	0.476	Eq	19	1	N/A
4016	10	>7.90	6.68	N/A	0	0.458	Eq	6	1	N/A
4026	13	6.60	5.51	0.081	0	0.981	Eq	8	1	N/A
4029	9	5.79	3.42	0.004	0	0.17	Eq, Amp	8	1	N/A
4032	32	5.23	3.71	0.031	0	0.578	Eq, Amp	29	1	N/A
4037	12	6.23	3.18	0.001	0	0.821	Eq	15	1	N/A
4038	16	6.40	4.05	0.004	0	0.771	Eq	11	1	N/A
4039	3	5.70	1.75	0.000	0	0.203	Eq	7	1	N/A
4041	5	4.85	4.55	0.497	0	0.259	Eq	6	1	N/A
4045	20	6.77	4.74	0.009	0	0.062	Eq	19	1	N/A
4046	16	6.39	4.95	0.036	0	0.943	Eq	15	1	N/A
4055	9	6.67	4.46	0.006	22	0.139	Eq	71	2	*
4056	22	6.36	4.02	0.005	0	0.501	Eq	16	1	N/A
4059	28	5.57	4.59	0.105	0	0.269	Eq	32	1	N/A
3017	5	7.04	4.83	0.006	38	0.091	Inter	24	2	*
3037	8	6.82	4.54	0.005	39	0.095	Inter, Amp	6	1	4
4007	16	5.79	3.72	0.008	33	0.179	Inter, Amp	24	1	16
4008	16	6.43	4.58	0.014	41	0.082	Inter	18	1	14
4009	14	6.23	3.85	0.004	25	0.210	Inter	10	1	8
4013	6	5.57	3.48	0.008	28	0.0001^g^	Inter, Amp	7	1	4
4028	17	5.70	3.72	0.010	40	0.157	Inter	6	1	5
4034	21	6.36	4.27	0.008	67	0.171	Inter	15	1	8
4036	12	6.71	3.82	0.001	75	0.068	Inter	4	1	4
4048	18	5.61	2.94	0.002	39	0.448	Inter, Amp	53	>2	*
4060	12	6.66	3.78	0.001	24	0.282	Inter	19	1	13
3002	5	6.48	5.07	0.039	71	0.008	Comp	57	2	8
3009	17	3.64	3.35	0.512	68	<0.0001	Comp	22	1	6
4002	21	5.99	4.15	0.015	45	0.001	Comp	19	1	12
4004	34	5.87	4.97	0.126	95	<0.0001	Comp, Amp	52, 47^j^	≥3	52, 24
4017	28	6.02	4.64	0.041	74	0.0001	Comp	26	1	19
4027	35	5.53	3.47	0.009	73	0.0002	Comp, Amp	21	1	7
4030	17	5.84	4.67	0.067	90	<0.0001	Comp	15	1	10
4031	26	4.76	2.80	0.011	67	0.001	Comp	11	1	5
4049	18	7.08	6.32	0.174	94	<0.0001	Comp	16	1	9
4050	23	6.01	3.98	0.009	65	0.024	Comp	17	1	12
4058	20	5.52	4.13	0.041	40	<0.0001	Comp, Amp	18	1	5
4061	19	5.52	3.22	0.005	65	0.013	Comp	9	1	4

a,b VL, viral load; HIV-1 RNA (log_10_ copies/ml).

c CSF/blood VL ratio.

d The percent of CSF *env* sequences that were compartmentalized (comp.) for each subject. CSF *env* sequences were considered compartmentalized when ≥4 sequences were found within the same clade and bootstrap values ≥40 were observed.

e *P* values used to measure genetic compartmentalization between the blood plasma and CSF HIV-1 populations were obtained using the Slatkin-Maddison test for gene flow between populations [31]. A *P* value<0.05 indicated statistically significant genetic compartmentalization.

f *P* value<0.05 was obtained for subject 3040, but visual assessment of the neighbor-joining tree structure indicated the presence of an additional HIV-1 population within the blood plasma that was largely absent from the CSF.

g *P* value<0.05 was obtained for subject 4013, but visual assessment of neighbor-joining tree structure indicated significant compartmentalization was not present.

h HIV-1 population characteristics in the CSF compartment (compart). Eq, equilibrated blood plasma and CSF populations; Inter (Intermediate), a minor subpopulation of the CSF was compartmentalized; Comp, significant compartmentalization in the CSF; Amp, clonal amplification of ≥3 variants detected in the CSF; N/A, not applicable when the CSF viral load was too low to obtain enough CSF *env* sequences.

i The time to most recent common ancestor (TMRCA) analyzed by Bayesian Evolutionary Analysis by Sampling Trees (BEAST) [33].

j For patient 4004, ≥3 transmitted variants observed: the first variant evolved at 52 months; the second and third variants evolved at 47 months.

k Number of variants transmitted.

l TMRCA for compartmentalized (Comp) population for intermediate and compartmentalized subjects. Equilibrated subjects were not analyzed (N/A). When recombination occurred between transmitted variants, BEAST was unable to date TMRCA (*).

Compartmentalization was assessed visually and statistically using the Slatkin-Maddison test [31]. CNS compartmentalization was defined by a Slatkin-Maddison *P* value<0.05 and a genetically distinct CSF population with a bootstrap value ≥40; we used a relatively low bootstrap value in this exploratory analysis because of the overall low diversity of the viral population in these children. Intermediate populations were defined by a Slatkin-Maddison *P* value>0.05 but with visual evidence of a minor CSF subpopulation of ≥4 CSF amplicons and a bootstrap value ≥40. Equilibrated populations were defined by a Slatkin-Maddison *P* value>0.05 and no evidence of a minor or major CSF population (Table 1).

Phylogenetic trees were also examined for clonal amplification. Clonally amplified lineages were defined as having short branch lengths in the neighbor-joining phylogenetic tree with bootstrap values ≥99 and a clade of ≥3 variants. These lineages signify the recent amplification of identical or nearly identical variants.

### Relationship of viral populations in blood and CSF

**Figure 1:**
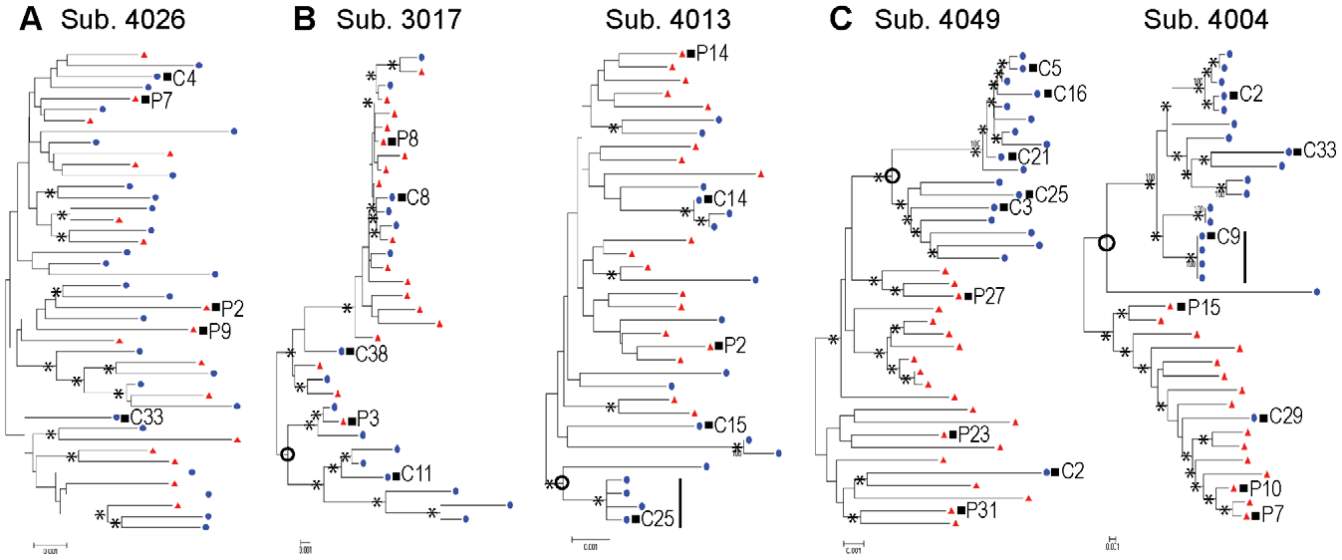
HIV-1 blood and CSF populations in infected children can be well equilibrated, intermediate or compartmentalized. Neighbor-joining phylogenetic trees. *env* sequences from the CSF are labeled with solid blue circles and *env* sequences from the blood plasma are labeled with solid red triangles. Bootstrap values ≥40 are indicated (*) at the appropriate nodes. Genetic distance is indicated at the bottom of each figure (0.001) and indicates the number of nucleotide substitutions per site between *env* sequences. Compartmentalized populations are indicated by open circle. Clonal amplification is indicated by solid bar. (a) One representative subject with well equilibrated blood plasma and CSF populations. (b) Two subjects demonstrating an intermediate condition between the blood plasma and CSF viral population. (c) Two subjects demonstrating the presence of a statistically significant compartmentalized population within the CSF relative to the blood, as assessed using the Slatkin-Maddison test [31].

Twenty out of 43 subjects (46%) had equilibrated viral populations in their blood and CSF (Table 1 and Figure 1A). For these subjects, sequences from the two compartments were well mixed and the CSF sequences were not genetically distinct from those of the blood. Two of these subjects did, however, display evidence of minor clonal amplification in the CSF.

In 11 out of 43 subjects (26%), an intermediate condition existed where the peripheral blood and CSF HIV-1 populations were not uniformly equilibrated and contained a minor CSF subpopulation (Table 1 and Figure 1B). Six of these intermediate subjects displayed evidence of clonal amplification. We hypothesize that the minor CSF population may indicate a precursor population within the CNS with the potential to expand into a compartmentalized population as infection progresses (see relationship with age below).

Significant genetic compartmentalization was detected between the blood and CSF populations in 12 out of 43 subjects (28%) (Table 1 and Figure 1C) indicating the presence of an independent, autonomously replicating viral population within the CNS. In these subjects, the virus in the CSF was genetically distinct from the virus in the blood. In 5 compartmentalized subjects, evidence of clonal amplification was observed. Therefore, within the first three years of HIV-1 subtype C pediatric infection, significant genetic compartmentalization can be observed.

### Compartmentalization is significantly related to older age and a higher CSF/blood viral load ratio

**Figure 2:**
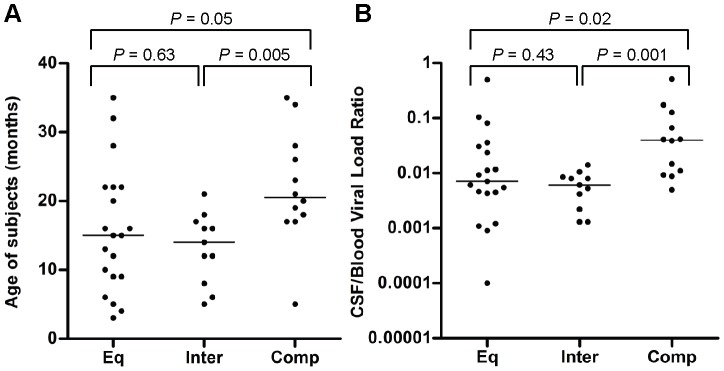
Older age and a higher CSF/blood viral load ratio are strong determining factors for compartmentalization. Relationships between clinical characteristics and genetic compartmentalization were assessed using the Mann-Whitney test in GraphPad Prism 4. Horizontal bars represent median values. A *P* value<0.05 was considered statistically significant. (a) Comparisons between age of the subjects and compartmentalization. (b) Comparisons between the CSF/blood viral load ratio of the subjects and compartmentalization.

Relationships between subject characteristics and compartmentalization were assessed using the Mann-Whitney test. Older children were more likely to have compartmentalized CSF variants when compared to equilibrated (*P* = 0.05) and intermediate subjects (*P* = 0.005) (Figure 2A). As the majority of vertical transmission occurs early, either *in utero*, at delivery or during the first months of breast feeding [32], older age provides a longer period of time for viral variants to become established within the CSF. No relationship was observed between the blood and CSF viral loads and subject classifications (Figure S1). However, a higher CSF/blood viral load ratio was significantly related to compartmentalization when compared to equilibrated (*P* = 0.02) or intermediate subjects (*P* = 0.001) (Figure 2B), consistent with the occurrence of local replication and expansion of viral populations within the CNS independent from the peripheral blood. Thus, compartmentalization most often appears subsequent to transmission and is associated with a higher CSF/blood viral load ratio, representing virus produced locally in the CNS.

### Evolutionary history of viral populations within the first three years of life

**Figure 3:**
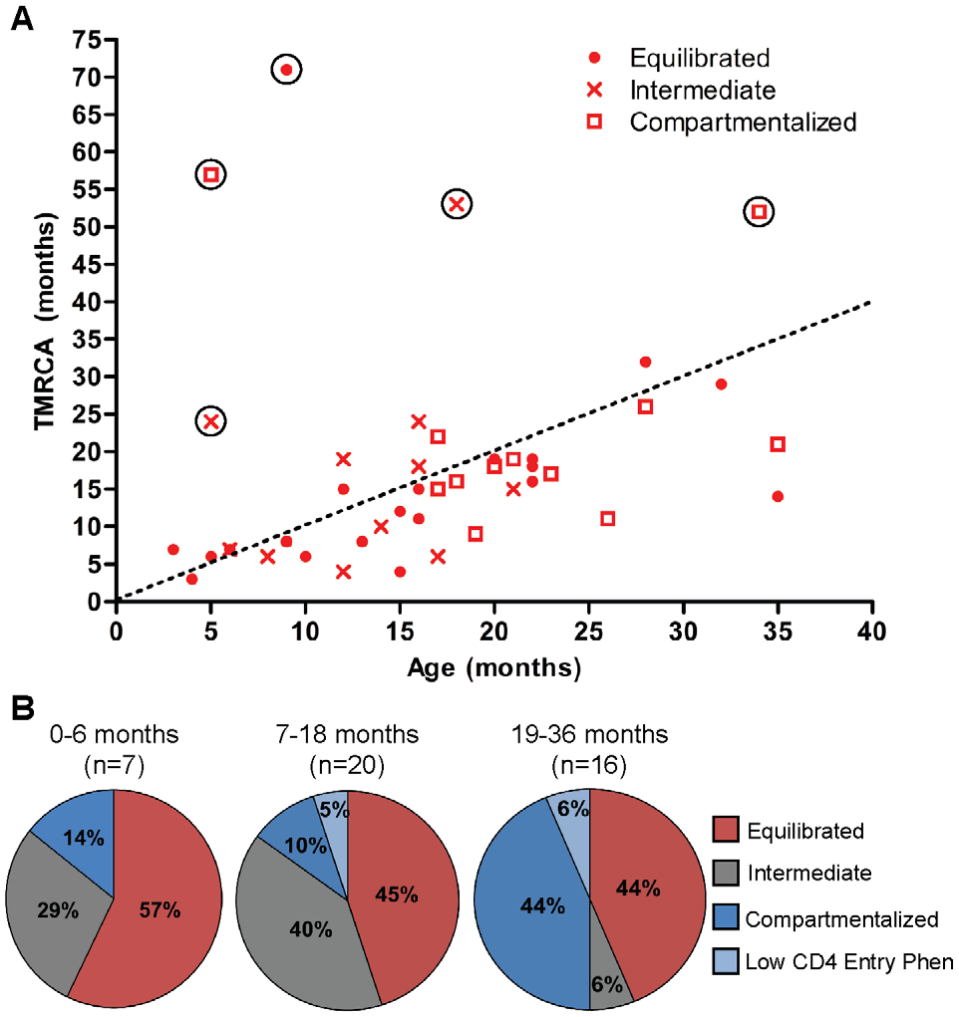
Evolutionary history of HIV-1 populations within the first three years of pediatric CNS infection. Age and TMRCA comparisons over the first three years of life. (a) Relationship between age of subjects and TMRCA of plasma population determined by BEAST [33]. Concordance of age and TMRCA indicated by dotted line. TMRCA outliers are indicated by open circle; equilibrated, subject 4055; intermediate, subjects 3017 and 4048; compartmentalized, subjects 3002 and 4004. (b) Pie charts grouping the children by age and showing the percentage of children in each state as a function of time. Phen, phenotype.

Bayesian Evolutionary Analysis by Sampling Trees (BEAST) [33] was used to estimate the time to most recent common ancestor (TMRCA) of the entire viral populations and the compartmentalized populations (Table 1). For the majority of the subjects, transmission was predicted to have occurred at birth ±6 months, as shown by the good concordance between the TMRCA and the age of the child at the time of sampling (Figure 3A). For 5 subjects, the predicted TMRCA was substantially higher than the age of the child, which, based on further analysis (discussed below), was probably due to multiple transmitted viruses.

As the majority of subjects were probably infected at or around the time of birth, we were able to depict the occurrence of compartmentalization as a function of time (Figure 3B). Before age 18 months, the populations in half the children were equilibrated, with more intermediate populations than compartmentalized in the remaining half. After age 18 months, about half of the children continued to have equilibrated populations while in the remainder, compartmentalized populations were now much more prevalent than intermediate populations. These data further support a potential transition over time in one-half of the children to increasing CNS compartmentalization in the absence of antiretroviral therapy.

### Evidence of multiple transmitted variants

**Figure 4:**
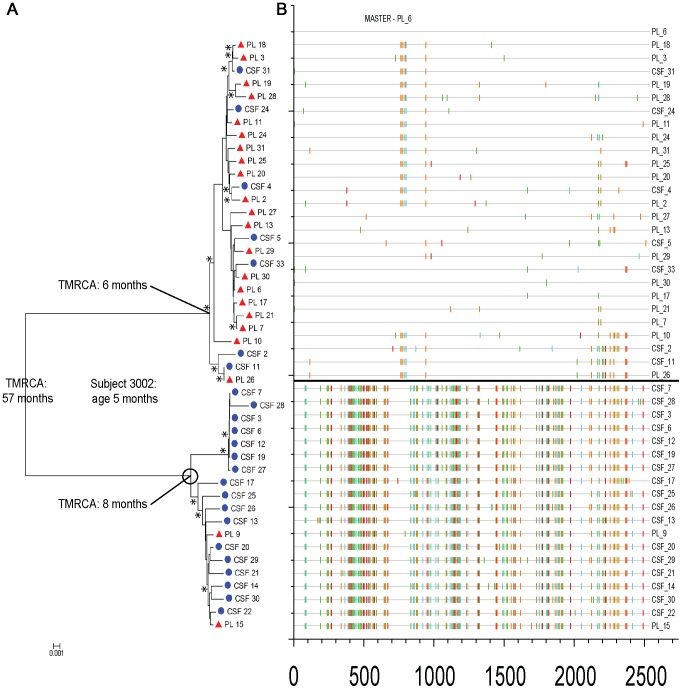
Evidence of multiple transmission events during pediatric HIV-1 subtype C infection. Phylogenetic and sequence analysis of plasma and CSF HIV-1 populations for subject 3002. (a) Neighbor-joining tree. Sequences from the CSF are labeled with solid blue circles, and plasma sequences (PL) are labeled with solid red triangles. Bootstrap values ≥40 are indicated (*) at the appropriate nodes. Genetic distance is scaled at the bottom of the figure (0.001) and indicates the number of nucleotide substitutions per site between *env* sequences. The subject's age is noted, as well as the overall TMRCA and the TMRCA of the two transmitted viruses. The CNS sequestered population is represented by an open black circle. (b) Highlighter plot of aligned *env* plasma and CSF sequences, generated at www.hiv.lanl.gov. The HXB2 nucleotide position number is indicated on the *x* axis, and the sequence identifier is indicated on the *y* axis. Nucleotide changes are indicated by the following ticks on the highlighter plot: A, green; T, red; G, orange; and C, blue. The two transmitted populations are separated by a heavy black line.

For 5 subjects for whom the predicted TMRCA was substantially greater than the age of the subject (Figure 3A), further analysis revealed the presence of multiple transmitted viruses (Table 1). For one compartmentalized subject (3002; age 5 months), phylogenetic analysis revealed a deep bifurcation separating two distinct viral populations, one comprised almost exclusively of CSF variants, and the other comprised primarily of blood variants (Figure 4A). Sequence analysis demonstrated that the viral populations were genetically distinct and had minimal recombination (Figure 4B). The overall TMRCA was 52 months, while the TMRCAs for the distinct plasma and CSF populations were 6 and 8 months, respectively. Together, these results indicate that the mother likely transmitted two genetically distinct viruses to the child, and one variant was sequestered within the CNS while the other was maintained within the periphery. A multiple variant transmission event was observed in one additional compartmentalized subject (Figure S2), two intermediate subjects (Figure S3 and S4) as well as one equilibrated subject (Figure S5). For the compartmentalized and intermediate subjects, one virus was sequestered within the CNS. For the equilibrated subject, both transmitted variants expanded within the blood and CSF. These data suggest that some variants can get selectively established in the CNS early after transmission.

### Evolution of CSF viruses to infect cells with low levels of CD4 surface expression

**Figure 5:**
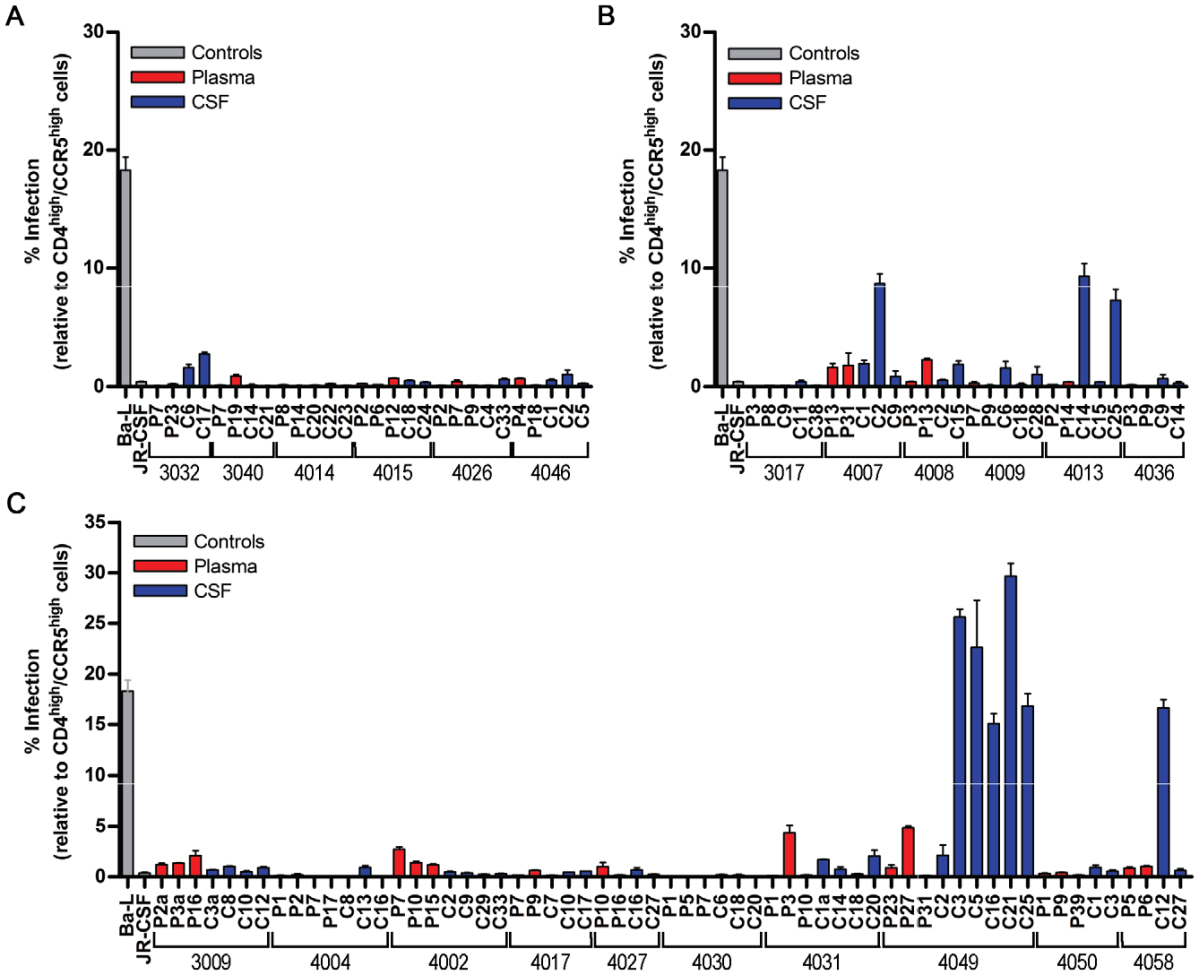
Compartmentalized HIV-1 CSF populations in children can have a low CD4 entry phenotype. Single-cycle infection of HIV-1 Env-pseudotyped reporter viruses on CD4^low^CCR5^high^ 293-Affinofile cells [34]. Receptor expression was measured as follows: CD4^low^ = 1,702 receptors/cell, CD4^high^ = 63,387 receptors/cell, CCR5^high^ = 26,117 receptors/cell. The data are averaged from triplicate wells for each of 2 to 3 env clones that were generated per indicated amplicon. Amplicons were selected for cloning to represent different portions of the phylogenetic tree. Panels show infection results for: (a) equilibrated subjects; (b) intermediate; and (c) compartmentalized subjects. Selected HIV-1 *env* sequences are indicated by a black square followed by the *env* name on neighbor joining trees for several subjects (Figure 1).

Macrophage tropism of HIV-1 is associated with the ability to infect cells expressing low levels of CD4 [11], [12], [13], while R5 T cell-tropic viruses infect these cells very poorly and require high levels of CD4 to enter cells [18], [19]. However, different preparations of macrophages vary significantly in their ability to be infected due to differing levels of CD4 in separate preparations of monocyte-derived macrophages (MDM) (Joseph et al., in preparation). To avoid this confounding variability, we have turned to a cell line that has regulatable levels of CD4 and CCR5, i.e. 293-Affinofile cells [34]. Entry phenotype was assessed by measuring the ability of pseudotyped reporter viruses to enter cells expressing either high or low levels of CD4. Viruses pseudotyped with Env proteins derived from virus in equilibrated subjects were only able to infect Affinofile cells with high CD4 surface expression and were considered R5 T cell-tropic (Figure 5A). Viruses pseudotyped with Env proteins derived from virus in intermediate subjects were also only able to infect cells expressing high levels of CD4 which we infer defines R5 T cell tropism (Figure 5B). A partially evolved entry phenotype was observed in subjects 4007 and 4013, where CSF variants were able to infect cells with low CD4 at modest levels, potentially identifying a precursor population to the low CD4 entry phenotype. Examples of viruses with a low CD4 entry phenotype were observed in two compartmentalized subjects (4049 and 4058) (Figure 5C). For both of these subjects, only the Env-pseudotyped viruses derived from the compartmentalized CSF population, not virus from the blood, were able to infect cells with low CD4 surface expression. These results indicate that subtype C HIV-1 viruses with a low CD4 entry phenotype can be detected in the CSF of children, but this is not a common occurrence within the first three years. Thus, we hypothesize that in most children replication in the CNS is sustained by growth in T cells, while in a subset (10–20% of children with CNS compartmentalized virus) the virus evolves to replicate in cells with low CD4 surface expression, potentially macrophages and/or microglia.

## Discussion

Our study design involves cross-sectional sampling and thus has several limitations, especially with regard to inferring temporal relationship. However, there is a wide distribution of ages of the subjects within the enrollment criteria allowing us to compare between different age groups and to draw correlations based on age. Also, the virus carries the history of longitudinal evolution in its sequence, thus allowing us to infer dates of bottlenecks in the history of viral replication. While cross-sectional analyses are inherently limited, we have some basis for suggesting temporal relationships in the observed phenomenon. Based on our results we hypothesize four distinct states to describe the relationship between virus in the CSF/CNS and virus in the blood/periphery. The first state has no genetic evidence for HIV-1 replication in the CNS, wherein the only virus detected in the CSF is genetically similar to that in the blood and is typically present at 1% or lower of the level in the blood, possibly due to some import or spill pathway from the blood into the CSF. The second state occurs prior to 18 months of age and involves minor compartmentalization of the CSF viral population, suggesting some local replication in the CSF/CNS but not to a level where the viral load increases in the CSF. In 10–20% of these children there is evidence for the initial evolution of virus that can use lower levels of CD4, potentially on a path to becoming macrophage-tropic. The third state occurs in about half of the children older than 18 months of age, and in this state the viral population in the CSF shows strong evidence of genetic compartmentalization, indicative of local replication and evolution within the CNS. This is also accompanied by a higher relative viral load in the CSF due to the local production of virus well above the low level that is imported from the periphery. In about 10–20% of the children with compartmentalized virus in the CSF, we identified variants that had evolved to use low levels of CD4, which we presume indicates that the virus was now growing in macrophages and/or microglia within the CNS. The fourth state involves multiple variant transmissions from mother to infant of which one variant preferentially replicates in the CNS and another replicates in the periphery. As HIV-1 replication in the CNS can contribute to neurological disease, further research should determine whether the ability to detect different states of CSF viral populations within the CNS of young children could guide strategies to monitor and prevent neurodevelopmental disorders in HIV-infected children.

Vertically transmitted viruses are often highly homogeneous, representing infection seeded by a single variant and characterized by low diversity [35], [36], [37], [38]. We identified five infants who appeared to be infected with multiple variants, with the viral populations in the remaining infants having a phylogenetic age consistent with the age of the infant, which we assume indicates infection with a single variant. Surprisingly, in four of the five infants infected with multiple variants, one of the variants was largely sequestered in the CNS/CSF. For this to occur, either one of the transmitted variants had a selective tropism for the CNS, or infection of the CNS was a low probability event influenced by the chance introduction of a founder virus. Alternatively, since the CNS is a somewhat immune-privileged site, the absence of the sequestered virus in the periphery may be due to selection by maternal antibodies or the initial infant immune response in that compartment. All of the *env* genes tested from three of these subjects (3002, 3017, and 4002; see Figure 5) showed that the pseudotyped viruses required high levels of CD4 to enter cells, i.e. were R5 T cell-tropic. In our previous work we found multiple variants in approximately 30% of infants infected vertically [38], which is not substantially different from the number found here. The sequestration of virus in the CNS shortly after transmission suggests that inferring the number of transmitted variants based on the complexity of virus in the blood may result in an underestimate of the frequency of transmission of multiple variants.

We can infer several other features of compartmentalization by comparing the age of the infant to the inferred age of the viral population using BEAST for those infants infected with a single variant. In the remaining 10 infants with compartmentalization who were infected with a single variant, the age of the CSF/CNS compartmentalized viral population was significantly less than the age of the entire viral population when compared to the age of the infant (*P* = 0.002; Wilcoxon signed rank test). In these cases it appears that compartmentalization is established after the initial stages of infection, with the compartmentalized virus emerging on average at 13.5 months but with outgrowth in the CNS only becoming apparent in the CSF approximately 18 months after birth. Thus we can identify two distinct pathways to compartmentalization: early sequestration of a transmitted virus in the CNS, or the later establishment of independently replicating virus that originates in the periphery. The compartmentalized lineage in the intermediate group appeared earlier after birth (mean 5.1 months) compared to the compartmentalized lineage in the compartmentalized group (mean 13.5 months) (*P* = 0.008; Mann-Whitney test). This may be an indication that the intermediate state represents susceptibility to viral replication in the CNS but that there is a subsequent bottleneck that defines the CNS population.

Genetically compartmentalized R5 T cell-tropic and macrophage-tropic HIV-1 subtype B populations have been shown to be associated with neurological complications in adults [9]. The macrophage-tropic populations were genetically diverse, representing established CNS infections, while the R5 T cell-tropic populations were clonally amplified and associated with pleocytosis [9]. Macrophage-tropic HIV-1 variants are generally characterized by the ability to infect cells with low CD4 surface expression [11], [12], [13]. However, infection using MDM from healthy donors is highly variable, and the variability is correlated with different levels of CD4 (Joseph et al., in preparation). For this reason, it is more quantitative to use a cell line where the levels of CD4 are regulatable and reproducible. Thus we have used Affinofile cells [34] as a surrogate for the entry phenotype of viruses able to use low levels of CD4 versus those requiring high levels of CD4. Our results demonstrated that compartmentalized R5 T cell-tropic and what we infer to be macrophage-tropic populations can also be found in the CSF of children infected with HIV-1 subtype C. A partial entry phenotype was observed in two intermediate subjects; we hypothesize that this is evidence for the initial evolution of virus that can use lower levels of CD4, potentially on a path to becoming macrophage-tropic.

Viral replication in the CNS results in the local production of inflammatory and neuronal destruction molecules such as monocyte chemoattractant protein (MCP-1), neopterin, IP-10, and neurofilament light subunit (NFL). Production of these inflammatory markers has been observed in animal models [39], [40] and has been linked to HIV-1-associated neurocognitive damage in adults [41], [42], [43]. The potential for long term neurocognitive damage in children as a result of HIV-1-associated production of inflammatory markers within the CNS, our findings of compartmentalized viral replication with viral lineage established at 13.5 months on average, and the ability of a transmitted variant to become sequestered in the CNS shortly after transmission adds further justification to the policy of early initiation of antiretroviral treatment in children, in this case as part of an effort to prevent the establishment of compartmentalized viral populations that may contribute to neurological complications.

## Materials and Methods

### Study subject population

The study was approved by the Institutional Review Boards of the University of North Carolina at Chapel Hill and the University of Malawi College of Medicine in Blantyre. Permission to participate in the research study was obtained for all children through written informed consent by the caregiver. All subjects included in this study were HIV-1 subtype C-infected children between 3 and 35 months of age. HIV-1 infection was verified at time of enrollment by a positive PCR for HIV DNA/RNA if <18 months of age or two positive rapid HIV antibody tests after age 18 months. Samples were collected at a one pre-HAART baseline visit for all subjects. CSF and blood plasma samples were used for viral genetic compartmentalization and *env* protein phenotypic analyses. Blood plasma and CSF HIV-1 viral loads (copies/mL) were determined by the UNC Chapel Hill Center for AIDS Research Virology Core.

### Single genome amplification

Subtype C HIV-1 RNA was isolated from blood plasma and CSF samples as previously described [16]. Briefly, viral RNA was isolated from blood plasma and CSF samples (140 µL) using the QIAmp Viral RNA Mini kit (Qiagen). Prior to RNA isolation, all blood plasma and CSF samples were pelleted (0.1–0.5 mL) by centrifugation at 25,000×g for 1.5 hours at 4°C to increase template number and improve sampling. Purified viral RNA (10–50 µl) was reverse transcribed using Superscript III Reverse transcriptase (Invitrogen) and an oligo-d(T) primer according to the manufacturer's instructions. Single genome amplification (SGA) of the full-length HIV-1 *env* gene through the 3′ LTR U3 end was conducted as previously described [30]. Briefly, cDNA was endpoint diluted and nested PCR was completed using Platinum Taq High Fidelity polymerase (Invitrogen) and the primers Vif1 [30] and 2.R3.B6R (5′-TGAAGCACTCAAGGCAAGCTTTATTGAGGC-3′; nt 9607 to 9636), and EnvA [30] and Low2c (5′-TGAGGCTTAAGCAGTGGGTTCC-3′; nt 9591 to 9612), were used for the first and second rounds of PCR, respectively. PCR amplicons were sequenced from the start of *env* through *env* gp41, gp160 end (HXB2 numbering of positions 6110–8833). Chromatograms with double peaks, indicating amplification from more than one cDNA template, as well as sequences with frameshift mutations resulting in premature stop codons, were excluded from analysis.

### Phylogenetic analysis of *env* viral sequences

DNA sequences alignments of *env* genes were performed using ClustalW [44]. Sequences for each subject were codon aligned (MEGA 4.0) and phylogenetic trees were generated using neighbor-joining method (MEGA 4.0) [45]. Compartmentalization of viral sequences was assessed using the Slatkin-Maddison test [31] available through HyPhy [46] using 10,000 permutations. No contamination occurred between samples (Figure S6).

### Bayesian analysis

A Bayesian Markov Chain Monte Carlo (MCMC) approach, as implemented in BEAST v.1.6.1 [33], estimated TMRCA for each patient sample. A substitution rate of 3.5×10^−5^ substitutions/site/generation was fixed under a strict clock model, as determined by calculation of inter-patient percent difference in the plasma nucleotide sequence. The HKY nucleotide substitution model had estimated base frequencies and a gamma-distributed rate heterogeneity (4 gamma categories). A coalescent Bayesian Skyline tree prior with a Piecewise-constant skyline model was used (4 groups). The MCMC algorithm was run for 30 million generations, logging every 1000 and with a 10% burn-in. The results from at least two independent runs were combined, and the effective sample size for all estimates was >200. A generation time of 1.5 days was used for estimation of time to the MRCA [47].

### Construction of HIV-1 *env* clones

SGA amplicons, selected based on the subject's phylogenetic tree structure, were re-amplified from the first-round nested PCR product using the Phusion hot start high-fidelity DNA polymerase (Finnzymes) and the primers EnvAClon (5′ CACCGGCTTAGGCATCTCCTGTGGCAGGAAGAA-3′; nt 5950–5982) and EnvN [30] following the manufacturer's instructions. HIV-1 *env* amplicons were then gel purified using the Qiagen gel extraction kit (QIagen). Purified HIV-1 *env* genes (50 ng) were cloned into the pcDNA3.1D/V5-His-TOPO expression vector (invitrogen) using the pcDNA 3.1 directional TOPO expression kit (Invitrogen) and the entire cloning reaction (6 µl) was transformed into MAX Efficiency Stbl2 competent cells (50 µl) as per the manufacturer's instructions. Bacterial colonies were screened for correct insertion of the HIV-1 *env* gene using colony PCR, and DNA was extracted from 3–6 positive colonies using the Qiaprep spin miniprep kit (Qiagen).

### Cells

293T cells were cultured in Dulbecco's modified Eagle medium (DMEM) supplemented with 10% fetal bovine serum (FBS) and 100 mg/ml of penicillin and streptomycin. 293-Affinofile cells [34] were maintained in DMEM supplemented with 10% dialyzed FBS (12–14 kD dialyzed; Atlanta Biologicals) and 50 mg/ml blasticidin (D10F/B). The Affinofile cell line was generously provided by Dr. Ben-Hur Lee.

### Env-pseudotyped viruses

Each Env-pseudotyped luciferase reporter virus was generated using the Fugene 6 transfection reagent and protocol (Roche) to co-transfect 293T cells with an *env* expression vector and the pNL4-3.LucR-E- HIV-1 backbone (obtained from the NIH AIDS Research and Reference Reagent Program, Division of AIDS, NIAID, NIH). Prior to transfection, 293T cells were seeded at a density of 4.8×10^5^ cells/well in 6-well tissue culture plates coated with 10% poly-L-lysine. Transfection medium was replaced five hours post-transfection with fresh culture medium and the cells were incubated at 37°C for 48 hours, after which viral supernatants were filtered with 0.45 µM filters (Millipore) and stored at −80°C.

### 293-Affinofile cellular surface expression of CD4 and CCR5

293-Affinofile cell [34] CD4 and CCR5 receptor expression was induced with doxycycline (doxy; Invitrogen) and ponasterone A (ponA; Invitrogen), respectively, and induced as previously described [16]. Briefly, cells were induced with doxy (0 ng/ml or 6 ng/ml) and ponA (5 µM) for 18–24 hours at 37°C and receptor expression was measured using quantitative fluorescence-activated cytometry (qFACS) following staining with either phycoerythin (PE)-conjugated anti-human CD4 antibody (clone Q4120, BD Biosciences) or PE-conjugated mouse anti-human CCR5 antibody (clone 2D7, BD Biosciences). CD4 and CCR5 receptor levels were quantified using *QuantiBRITE* beads (BD Biosciences).

### Single-cycle infection of 293-Affinofile cells

Env-pseudotyped luciferase reporter viruses were first titered in triplicate in a 96-well plate format on 293-Affinofile cells [34] expressing the maximum induction levels for both CD4 (6 ng/ml doxy) and CCR5 (5 µM ponA) surface expression as previously described [16]. In order to ensure that each infection assay was performed within the linear range, we used the volume of each virus needed to produce 800,000 relative light units (RLUs) when used to infect Affinofile cells expressing the highest levels of CD4 and CCR5. Two days prior to infection, 96-well, black tissue culture plates were coated with 10% poly-L-lysine and then seeded with 293-Affinofile cells at a density of 1.85×10^4^ cells/well. 18–24 hours later, expression of CD4 and CCR5 was induced at two conditions in triplicate: CD4^high^/CCR5^high^ (6 ng/ml doxy and 5 µM ponA, respectively) and CD4^low^/CCR5^high^ (0 ng/ml doxy and 5 µM ponA). 18 to 24 hours later, the induction medium was removed and gently replaced with 100 µl of fresh, warmed culture medium containing *env*-pseudotyped virus. The infection plates were spinoculated [48] at 2,000 rpm for 2 hours at 37°C, and then incubated for an additional 48 hours at 37°C. Infection medium was then removed, the cells were lysed, and luciferase activity was assayed using the luciferase assay system (Promega).

### Nucleotide sequence accession numbers

The HIV-1 *env* nucleotide sequences determined in this study have been deposited in GenBank under accession numbers KC186127-KC187733.

## Supporting Information

Figure S1 **No relationship between viral load and subject classification.** Relationships between blood or CSF viral loads and virological classifications were assessed using the Mann-Whitney test in GraphPad Prism 4. Horizontal bars represent median values. (a) Comparisons between blood viral load and CSF compartment classification (Eq, equilibrated; Inter, Intermediate; Comp, compartmentalized.) (b) Comparisons between blood viral load and TMRCA classification. (c) Comparisons between CSF viral load and TMRCA classification.
(TIF)

Figure S2 **Compartmentalized subject 4004 exhibiting three or more transmitted viruses.** Phylogenetic and sequence analysis of plasma and CSF HIV-1 populations for subject 4004. (a) Neighbor-joining tree. CSF sequences are labeled with solid blue circles, and plasma sequences (PL) are labeled with solid red triangles. Bootstrap values ≥40 are indicated (*) at the appropriate nodes. Genetic distance is scaled at the bottom of the figure (0.001) and indicates the number of nucleotide substitutions per site between *env* sequences. The subject's age is noted, as well as the TMRCA for the transmitted and compartmentalized populations. The CNS sequestered population is represented by an open black circle. (b) Highlighter plot of aligned *env* plasma and CSF sequences, generated at www.hiv.lanl.gov. The HXB2 base number is indicated on the *x* axis, and the sequence identifier is indicated on the *y* axis. Base changes are indicated by the following ticks on the highlighter plot: A, green; T, red; G, orange; and C, blue. Sequestered populations resulting from the transmitted viruses are separated by heavy black lines. One variant was sequestered within the CSF (top) and additional variants were established within the blood (middle). Another variant was also isolated from the CSF (bottom), but the population was not maintained.
(TIF)

Figure S3 **Intermediate subject 3017 exhibiting two transmitted viruses.** Phylogenetic and sequence analysis of plasma and CSF HIV-1 populations for subject 3017. (a) Neighbor-joining tree. Sequences from the CSF are labeled with solid blue circles, and plasma sequences (PL) are labeled with solid red triangles. Bootstrap values ≥40 are indicated (*) at the appropriate nodes. Genetic distance is scaled at the bottom of the figure (0.001) and indicates the number of nucleotide substitutions per site between *env* sequences. The subject's age is noted, as well as the overall TMRCA. BEAST was unable to assign a TMRCA to the internal nodes due to recombination in the population. The CNS sequestered population is represented by an open black circle. (b) Highlighter plot of aligned *env* plasma and CSF sequences, generated at www.hiv.lanl.gov. The highlighter plot characteristics are the same as those stated in Figure S2. Subject was likely infected with two viral variants during transmission. The two sequences that are closest to the parental strains are PL_14 (top) and CSF_3 (bottom), and recombination between the transmitted viruses appears to account for much of the *env* genetic diversity detected in both the plasma and CSF populations. The transmitted CSF variant was maintained within the CNS, generating a minor CSF population with small local replication, accounting for the intermediate state observed for this subject.
(TIF)

Figure S4 **Intermediate subject 4048 exhibiting greater than two transmitted viruses.** Phylogenetic and sequence analysis of plasma and CSF HIV-1 populations for subject 4048. (a) Neighbor-joining tree. Sequences from the CSF are labeled with solid blue circles, and plasma sequences (PL) are labeled with solid red triangles. Bootstrap values ≥40 are indicated (*) at the appropriate nodes. The phylogenetic tree characteristics are the same as those stated in Figure S3. (b) Highlighter plot of aligned *env* plasma and CSF sequences, generated at www.hiv.lanl.gov. The highlighter plot characteristics are the same as those stated in Figure S2. Several unique motifs were observed around 1000 base pairs (top), indicating that there were potentially greater than 2 transmitted viruses. Recombination between the transmitted viruses appears to account for much of the *env* genetic diversity detected in both the plasma and CSF populations. The transmitted CSF variant was maintained within the CNS, generating a minor CSF population with small local replication, accounting for the intermediate state observed for this subject.
(TIF)

Figure S5 **Equilibrated subject 4055 exhibiting two transmitted viruses.** Phylogenetic and sequence analysis of plasma and CSF HIV-1 populations for subject 4055. (a) Neighbor-joining tree. Sequences from the CSF are labeled with solid blue circles, and plasma sequences (PL) are labeled with solid red triangles. Bootstrap values ≥40 are indicated (*) at the appropriate nodes. Genetic distance is scaled at the bottom of the figure (0.001) and indicates the number of nucleotide substitutions per site between *env* sequences. The subject's age is noted, as well as the overall TMRCA. (b) Highlighter plot of aligned *env* plasma and CSF sequences, generated at www.hiv.lanl.gov. The highlighter plot characteristics are the same as those stated in Figure S2. Subject was likely infected with 2 viral variants during transmission. The two sequences that are likely closet to the parental strains are CSF_16 and PL_4, and recombination between the transmitted viruses appears to account for much of the *env* genetic diversity detected in both the plasma and CSF populations. Both variants were maintained within the blood and CSF, accounting for the equilibrated state observed for this patient.
(TIF)

Figure S6 **No contamination was observed between subjects.** Neighbor-joining phylogenetic tree (radial topology). *env* sequences from the CSF are labeled with solid blue circles and *env* sequences from the blood plasma are labeled with solid red triangles. Genetic distance is indicated at the bottom of the figure and indicates the number of nucleotide substitutions per site between *env* sequences. Each subject ID is indicated.
(TIF)

## References

[1] Schnell G, Price RW, Swanstrom R, Spudich S (2010) Compartmentalization and clonal amplification of HIV-1 variants in the cerebrospinal fluid during primary infection. J Virol 84: 2395–2407. 20015984 10.1128/JVI.01863-09 PMC2820937

[2] Ritola K, Robertson K, Fiscus SA, Hall C, Swanstrom R (2005) Increased human immunodeficiency virus type 1 (HIV-1) env compartmentalization in the presence of HIV-1-associated dementia. J Virol 79: 10830–10834. 16051875 10.1128/JVI.79.16.10830-10834.2005 PMC1182623

[3] Harrington PR, Schnell G, Letendre SL, Ritola K, Robertson K, et al (2009) Cross-sectional characterization of HIV-1 env compartmentalization in cerebrospinal fluid over the full disease course. AIDS 23: 907–915. 19414991 10.1097/QAD.0b013e3283299129 PMC3089803

[4] Ohagen A, Devitt A, Kunstman KJ, Gorry PR, Rose PP, et al (2003) Genetic and functional analysis of full-length human immunodeficiency virus type 1 env genes derived from brain and blood of patients with AIDS. J Virol 77: 12336–12345. 14581570 10.1128/JVI.77.22.12336-12345.2003 PMC254258

[5] Dunfee RL, Thomas ER, Gorry PR, Wang J, Taylor J, et al (2006) The HIV Env variant N283 enhances macrophage tropism and is associated with brain infection and dementia. Proc Natl Acad Sci U S A 103: 15160–15165. 17015824 10.1073/pnas.0605513103 PMC1586182

[6] Gorry PR, Bristol G, Zack JA, Ritola K, Swanstrom R, et al (2001) Macrophage tropism of human immunodeficiency virus type 1 isolates from brain and lymphoid tissues predicts neurotropism independent of coreceptor specificity. J Virol 75: 10073–10089. 11581376 10.1128/JVI.75.21.10073-10089.2001 PMC114582

[7] Koenig S, Gendelman HE, Orenstein JM, Dal Canto MC, Pezeshkpour GH, et al (1986) Detection of AIDS virus in macrophages in brain tissue from AIDS patients with encephalopathy. Science 233: 1089–1093. 3016903 10.1126/science.3016903

[8] Peters PJ, Bhattacharya J, Hibbitts S, Dittmar MT, Simmons G, et al (2004) Biological analysis of human immunodeficiency virus type 1 R5 envelopes amplified from brain and lymph node tissues of AIDS patients with neuropathology reveals two distinct tropism phenotypes and identifies envelopes in the brain that confer an enhanced tropism and fusigenicity for macrophages. J Virol 78: 6915–6926. 15194768 10.1128/JVI.78.13.6915-6926.2004 PMC421670

[9] Schnell G, Joseph S, Spudich S, Price RW, Swanstrom R (2011) HIV-1 replication in the central nervous system occurs in two distinct cell types. PLoS Pathog 7: e1002286. 22007152 10.1371/journal.ppat.1002286 PMC3188520

[10] Brown RJ, Peters PJ, Caron C, Gonzalez-Perez MP, Stones L, et al (2011) Intercompartmental recombination of HIV-1 contributes to env intrahost diversity and modulates viral tropism and sensitivity to entry inhibitors. J Virol 85: 6024–6037. 21471230 10.1128/JVI.00131-11 PMC3126287

[11] Rossi F, Querido B, Nimmagadda M, Cocklin S, Navas-Martin S, et al (2008) The V1–V3 region of a brain-derived HIV-1 envelope glycoprotein determines macrophage tropism, low CD4 dependence, increased fusogenicity and altered sensitivity to entry inhibitors. Retrovirology 5: 89. 18837996 10.1186/1742-4690-5-89 PMC2576352

[12] Thomas ER, Dunfee RL, Stanton J, Bogdan D, Taylor J, et al (2007) Macrophage entry mediated by HIV Envs from brain and lymphoid tissues is determined by the capacity to use low CD4 levels and overall efficiency of fusion. Virology 360: 105–119. 17084877 10.1016/j.virol.2006.09.036 PMC1890014

[13] Gorry PR, Taylor J, Holm GH, Mehle A, Morgan T, et al (2002) Increased CCR5 affinity and reduced CCR5/CD4 dependence of a neurovirulent primary human immunodeficiency virus type 1 isolate. J Virol 76: 6277–6292. 12021361 10.1128/JVI.76.12.6277-6292.2002 PMC136234

[14] Alexander M, Lynch R, Mulenga J, Allen S, Derdeyn CA, et al (2010) Donor and recipient envs from heterosexual human immunodeficiency virus subtype C transmission pairs require high receptor levels for entry. J Virol 84: 4100–4104. 20147398 10.1128/JVI.02068-09 PMC2849512

[15] Salazar-Gonzalez JF, Salazar MG, Keele BF, Learn GH, Giorgi EE, et al (2009) Genetic identity, biological phenotype, and evolutionary pathways of transmitted/founder viruses in acute and early HIV-1 infection. J Exp Med 206: 1273–1289. 19487424 10.1084/jem.20090378 PMC2715054

[16] Schnell G, Spudich S, Harrington P, Price RW, Swanstrom R (2009) Compartmentalized human immunodeficiency virus type 1 originates from long-lived cells in some subjects with HIV-1-associated dementia. PLoS Pathog 5: e1000395. 19390619 10.1371/journal.ppat.1000395 PMC2668697

[17] Haas DW, Clough LA, Johnson BW, Harris VL, Spearman P, et al (2000) Evidence of a source of HIV type 1 within the central nervous system by ultraintensive sampling of cerebrospinal fluid and plasma. AIDS Res Hum Retroviruses 16: 1491–1502. 11054262 10.1089/088922200750006010

[18] Wei X, Ghosh SK, Taylor ME, Johnson VA, Emini EA, et al (1995) Viral dynamics in human immunodeficiency virus type 1 infection. Nature 373: 117–122. 7529365 10.1038/373117a0

[19] Ho DD, Neumann AU, Perelson AS, Chen W, Leonard JM, et al (1995) Rapid turnover of plasma virions and CD4 lymphocytes in HIV-1 infection. Nature 373: 123–126. 7816094 10.1038/373123a0

[20] Rao VR, Sas AR, Eugenin EA, Siddappa NB, Bimonte-Nelson H, et al (2008) HIV-1 clade-specific differences in the induction of neuropathogenesis. J Neurosci 28: 10010–10016. 18829958 10.1523/JNEUROSCI.2955-08.2008 PMC2572723

[21] Satishchandra P, Nalini A, Gourie-Devi M, Khanna N, Santosh V, et al (2000) Profile of neurologic disorders associated with HIV/AIDS from Bangalore, south India (1989–96). Indian J Med Res 111: 14–23. 10793489

[22] Riedel D, Ghate M, Nene M, Paranjape R, Mehendale S, et al (2006) Screening for human immunodeficiency virus (HIV) dementia in an HIV clade C-infected population in India. J Neurovirol 12: 34–38. 16595372 10.1080/13550280500516500

[23] Gabuzda DH, Hirsch MS (1987) Neurologic manifestations of infection with human immunodeficiency virus. Clinical features and pathogenesis. Ann Intern Med 107: 383–391. 3039890 10.7326/0003-4819-107-2-383

[24] Vincent JBM, Bash M, Shanks D, Daigh D, Moriarty R, et al (1989) Neurologic symptoms as the initial presentation of HIV infection in pediatric patients. Int Conf AIDS 4–9 June 1989; Montreal, Quebec, Canada.

[25] Van Rie A, Harrington PR, Dow A, Robertson K (2007) Neurologic and neurodevelopmental manifestations of pediatric HIV/AIDS: a global perspective. Eur J Paediatr Neurol 11: 1–9. 17137813 10.1016/j.ejpn.2006.10.006

[26] Keele BF, Giorgi EE, Salazar-Gonzalez JF, Decker JM, Pham KT, et al (2008) Identification and characterization of transmitted and early founder virus envelopes in primary HIV-1 infection. Proc Natl Acad Sci U S A 105: 7552–7557. 18490657 10.1073/pnas.0802203105 PMC2387184

[27] Palmer S, Wiegand AP, Maldarelli F, Bazmi H, Mican JM, et al (2003) New real-time reverse transcriptase-initiated PCR assay with single-copy sensitivity for human immunodeficiency virus type 1 RNA in plasma. J Clin Microbiol 41: 4531–4536. 14532178 10.1128/JCM.41.10.4531-4536.2003 PMC254331

[28] Simmonds P, Balfe P, Ludlam CA, Bishop JO, Brown AJ (1990) Analysis of sequence diversity in hypervariable regions of the external glycoprotein of human immunodeficiency virus type 1. J Virol 64: 5840–5850. 2243378 10.1128/jvi.64.12.5840-5850.1990 PMC248744

[29] Abrahams MR, Anderson JA, Giorgi EE, Seoighe C, Mlisana K, et al (2009) Quantitating the multiplicity of infection with human immunodeficiency virus type 1 subtype C reveals a non-poisson distribution of transmitted variants. J Virol 83: 3556–3567. 19193811 10.1128/JVI.02132-08 PMC2663249

[30] Salazar-Gonzalez JF, Bailes E, Pham KT, Salazar MG, Guffey MB, et al (2008) Deciphering human immunodeficiency virus type 1 transmission and early envelope diversification by single-genome amplification and sequencing. J Virol 82: 3952–3970. 18256145 10.1128/JVI.02660-07 PMC2293010

[31] Slatkin M, Maddison WP (1989) A cladistic measure of gene flow inferred from the phylogenies of alleles. Genetics 123: 603–613. 2599370 10.1093/genetics/123.3.603 PMC1203833

[32] Ahmad N (2005) The vertical transmission of human immunodeficiency virus type 1: molecular and biological properties of the virus. Crit Rev Clin Lab Sci 42: 1–34. 15697169 10.1080/10408360490512520

[33] Drummond AJ, Rambaut A (2007) BEAST: Bayesian evolutionary analysis by sampling trees. BMC Evol Biol 7: 214. 17996036 10.1186/1471-2148-7-214 PMC2247476

[34] Johnston SH, Lobritz MA, Nguyen S, Lassen K, Delair S, et al (2009) A quantitative affinity-profiling system that reveals distinct CD4/CCR5 usage patterns among human immunodeficiency virus type 1 and simian immunodeficiency virus strains. J Virol 83: 11016–11026. 19692480 10.1128/JVI.01242-09 PMC2772777

[35] Ahmad N, Baroudy BM, Baker RC, Chappey C (1995) Genetic analysis of human immunodeficiency virus type 1 envelope V3 region isolates from mothers and infants after perinatal transmission. J Virol 69: 1001–1012. 7815476 10.1128/jvi.69.2.1001-1012.1995 PMC188669

[36] Scarlatti G, Leitner T, Halapi E, Wahlberg J, Marchisio P, et al (1993) Comparison of variable region 3 sequences of human immunodeficiency virus type 1 from infected children with the RNA and DNA sequences of the virus populations of their mothers. Proc Natl Acad Sci U S A 90: 1721–1725. 8446584 10.1073/pnas.90.5.1721 PMC45951

[37] Mulder-Kampinga GA, Simonon A, Kuiken CL, Dekker J, Scherpbier HJ, et al (1995) Similarity in env and gag genes between genomic RNAs of human immunodeficiency virus type 1 (HIV-1) from mother and infant is unrelated to time of HIV-1 RNA positivity in the child. J Virol 69: 2285–2296. 7884875 10.1128/jvi.69.4.2285-2296.1995 PMC188899

[38] Russell ES, Kwiek JJ, Keys J, Barton K, Mwapasa V, et al (2011) The genetic bottleneck in vertical transmission of subtype C HIV-1 is not driven by selection of especially neutralization-resistant virus from the maternal viral population. J Virol 85: 8253–8262. 21593171 10.1128/JVI.00197-11 PMC3147968

[39] Harrington PR, Connell MJ, Meeker RB, Johnson PR, Swanstrom R (2007) Dynamics of simian immunodeficiency virus populations in blood and cerebrospinal fluid over the full course of infection. J Infect Dis 196: 1058–1067. 17763329 10.1086/520819

[40] Mankowski JL, Queen SE, Clements JE, Zink MC (2004) Cerebrospinal fluid markers that predict SIV CNS disease. J Neuroimmunol 157: 66–70. 15579282 10.1016/j.jneuroim.2004.08.031

[41] Cinque P, Bestetti A, Marenzi R, Sala S, Gisslen M, et al (2005) Cerebrospinal fluid interferon-gamma-inducible protein 10 (IP-10, CXCL10) in HIV-1 infection. J Neuroimmunol 168: 154–163. 16091292 10.1016/j.jneuroim.2005.07.002

[42] Hagberg L, Cinque P, Gisslen M, Brew BJ, Spudich S, et al (2010) Cerebrospinal fluid neopterin: an informative biomarker of central nervous system immune activation in HIV-1 infection. AIDS Res Ther 7: 15. 20525234 10.1186/1742-6405-7-15 PMC2890504

[43] Eden A, Price RW, Spudich S, Fuchs D, Hagberg L, et al (2007) Immune activation of the central nervous system is still present after >4 years of effective highly active antiretroviral therapy. J Infect Dis 196: 1779–1783. 18190258 10.1086/523648

[44] Thompson JD, Higgins DG, Gibson TJ (1994) CLUSTAL W: improving the sensitivity of progressive multiple sequence alignment through sequence weighting, position-specific gap penalties and weight matrix choice. Nucleic Acids Res 22: 4673–4680. 7984417 10.1093/nar/22.22.4673 PMC308517

[45] Tamura K, Dudley J, Nei M, Kumar S (2007) MEGA4: Molecular Evolutionary Genetics Analysis (MEGA) software version 4.0. Mol Biol Evol 24: 1596–1599. 17488738 10.1093/molbev/msm092

[46] Pond SL, Frost SD, Muse SV (2005) HyPhy: hypothesis testing using phylogenies. Bioinformatics 21: 676–679. 15509596 10.1093/bioinformatics/bti079

[47] Markowitz M, Louie M, Hurley A, Sun E, Di Mascio M, et al (2003) A novel antiviral intervention results in more accurate assessment of human immunodeficiency virus type 1 replication dynamics and T-cell decay in vivo. J Virol 77: 5037–5038. 12663814 10.1128/JVI.77.8.5037-5038.2003 PMC152136

[48] O'Doherty U, Swiggard WJ, Malim MH (2000) Human immunodeficiency virus type 1 spinoculation enhances infection through virus binding. J Virol 74: 10074–10080. 11024136 10.1128/jvi.74.21.10074-10080.2000 PMC102046

