# Supplementary figures and images for: Central Nervous System Compartmentalization of HIV-1 Subtype C Variants Early and Late in Infection in Young Children
Source: PLoS Pathog. 2012 Dec 27;8(12):e1003094. doi: 10.1371/journal.ppat.1003094 (PMC3531524; doi:10.1371/journal.ppat.1003094)

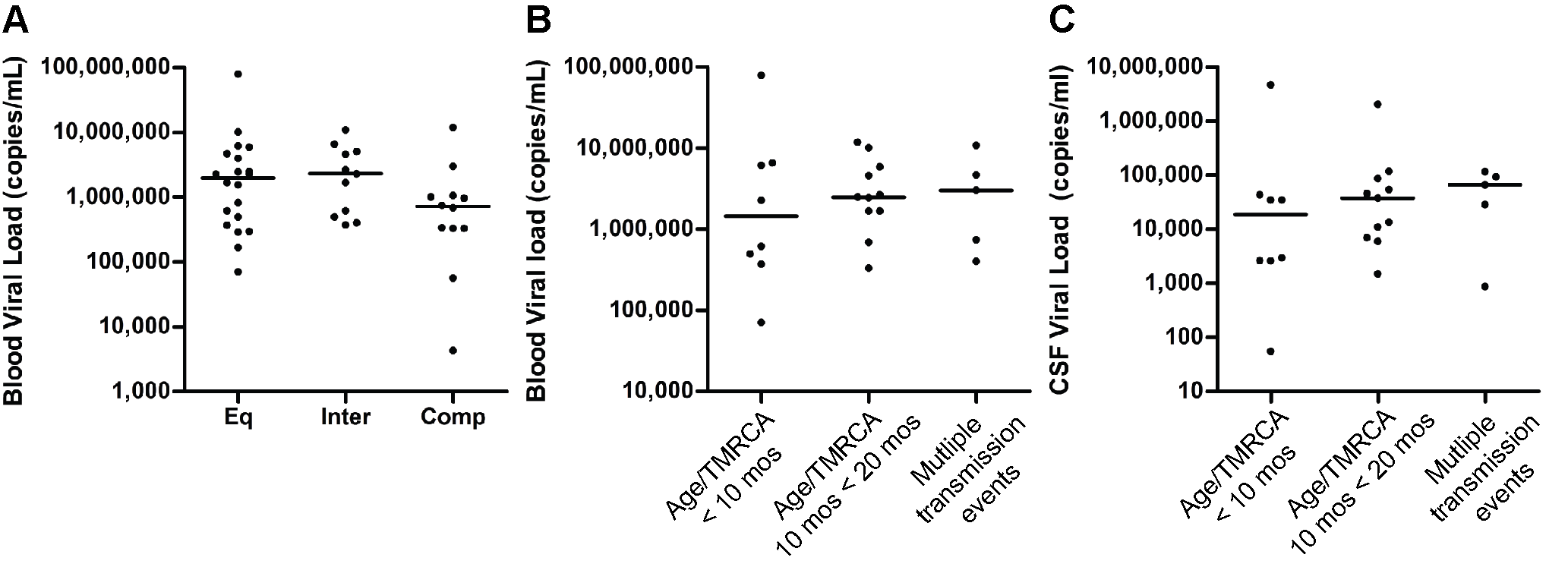

Supplement: Figure S1 — No relationship between viral load and subject classification. Relationships between blood or CSF viral loads and virological classifications were assessed using the Mann-Whitney test in GraphPad Prism 4. Horizontal bars represent median values. (a) Comparisons between blood viral load and CSF compartment classification (Eq, equilibrated; Inter, Intermediate; Comp, compartmentalized.) (b) Comparisons between blood viral load and TMRCA classification. (c) Comparisons between CSF viral load and TMRCA classification. (TIF) [file ppat.1003094.s001.tif]

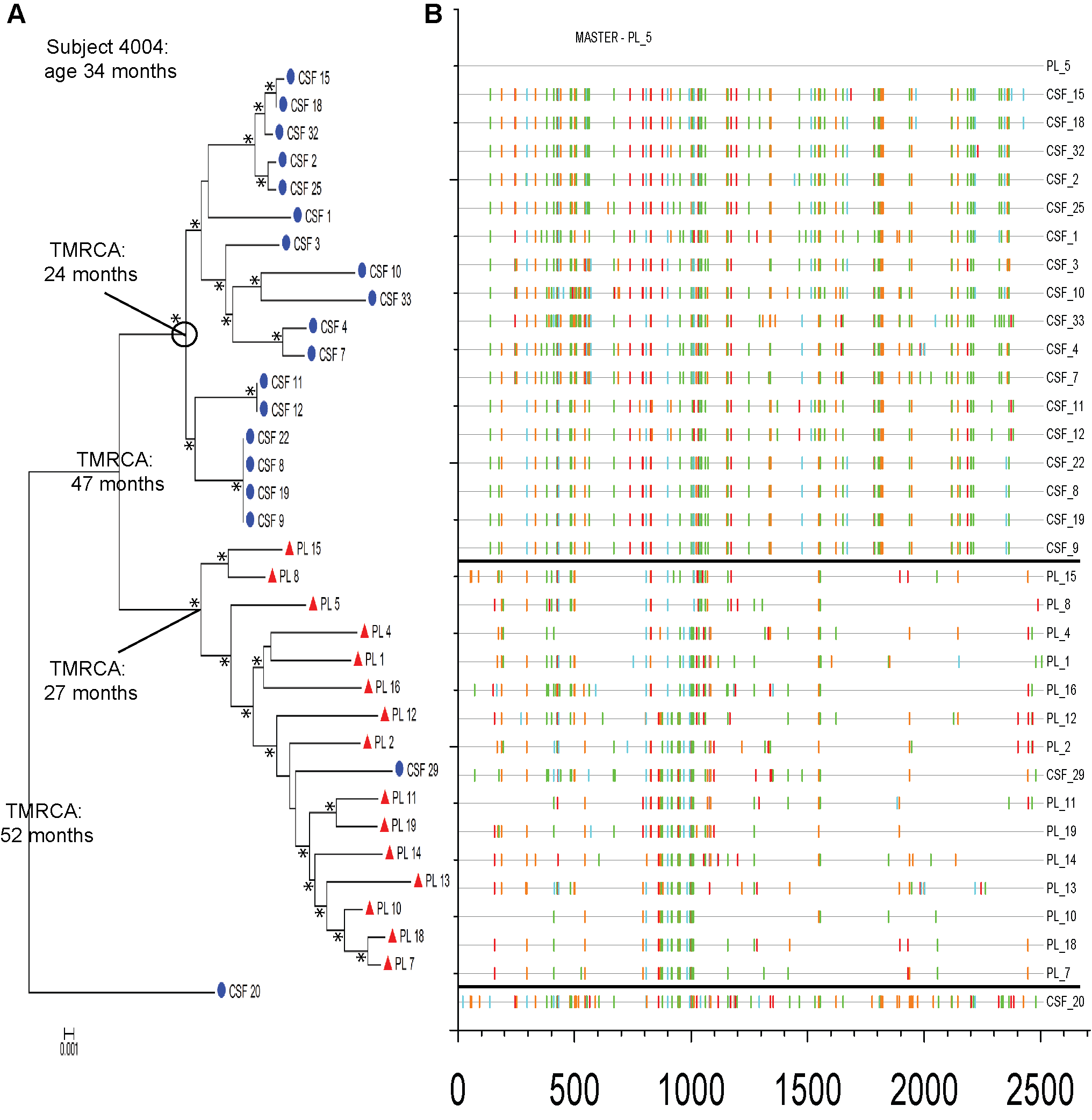

Supplement: Figure S2 — Compartmentalized subject 4004 exhibiting three or more transmitted viruses. Phylogenetic and sequence analysis of plasma and CSF HIV-1 populations for subject 4004. (a) Neighbor-joining tree. CSF sequences are labeled with solid blue circles, and plasma sequences (PL) are labeled with solid red triangles. Bootstrap values ≥40 are indicated (*) at the appropriate nodes. Genetic distance is scaled at the bottom of the figure (0.001) and indicates the number of nucleotide substitutions per site between env sequences. The subject's age is noted, as well as the TMRCA for the transmitted and compartmentalized populations. The CNS sequestered population is represented by an open black circle. (b) Highlighter plot of aligned env plasma and CSF sequences, generated at www.hiv.lanl.gov. The HXB2 base number is indicated on the x axis, and the sequence identifier is indicated on the y axis. Base changes are indicated by the following ticks on the highlighter plot: A, green; T, red; G, orange; and C, blue. Sequestered populations resulting from the transmitted viruses are separated by heavy black lines. One variant was sequestered within the CSF (top) and additional variants were established within the blood (middle). Another variant was also isolated from the CSF (bottom), but the population was not maintained. (TIF) [file ppat.1003094.s002.tif]

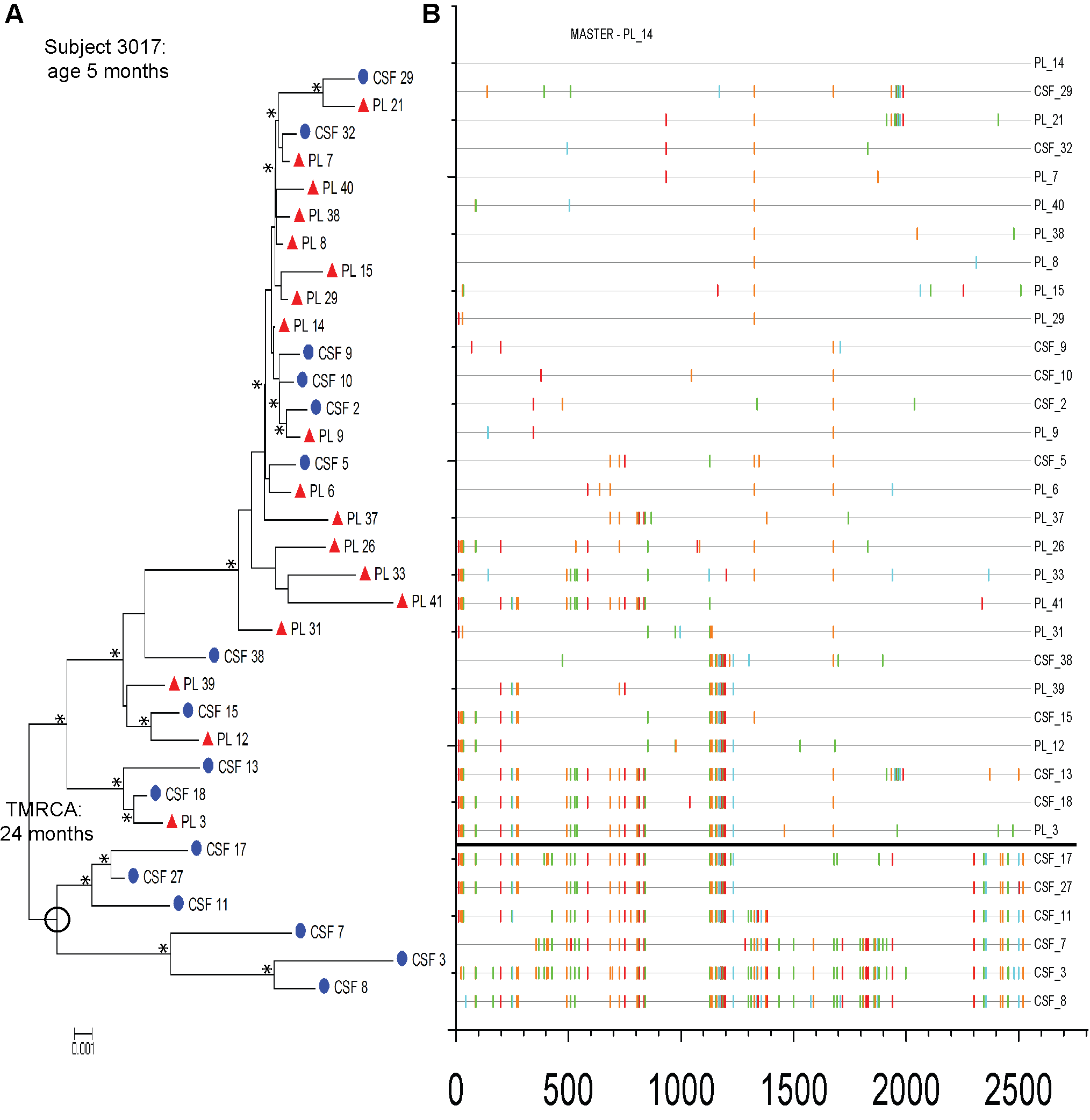

Supplement: Figure S3 — Intermediate subject 3017 exhibiting two transmitted viruses. Phylogenetic and sequence analysis of plasma and CSF HIV-1 populations for subject 3017. (a) Neighbor-joining tree. Sequences from the CSF are labeled with solid blue circles, and plasma sequences (PL) are labeled with solid red triangles. Bootstrap values ≥40 are indicated (*) at the appropriate nodes. Genetic distance is scaled at the bottom of the figure (0.001) and indicates the number of nucleotide substitutions per site between env sequences. The subject's age is noted, as well as the overall TMRCA. BEAST was unable to assign a TMRCA to the internal nodes due to recombination in the population. The CNS sequestered population is represented by an open black circle. (b) Highlighter plot of aligned env plasma and CSF sequences, generated at www.hiv.lanl.gov. The highlighter plot characteristics are the same as those stated in Figure S2. Subject was likely infected with two viral variants during transmission. The two sequences that are closest to the parental strains are PL_14 (top) and CSF_3 (bottom), and recombination between the transmitted viruses appears to account for much of the env genetic diversity detected in both the plasma and CSF populations. The transmitted CSF variant was maintained within the CNS, generating a minor CSF population with small local replication, accounting for the intermediate state observed for this subject. (TIF) [file ppat.1003094.s003.tif]

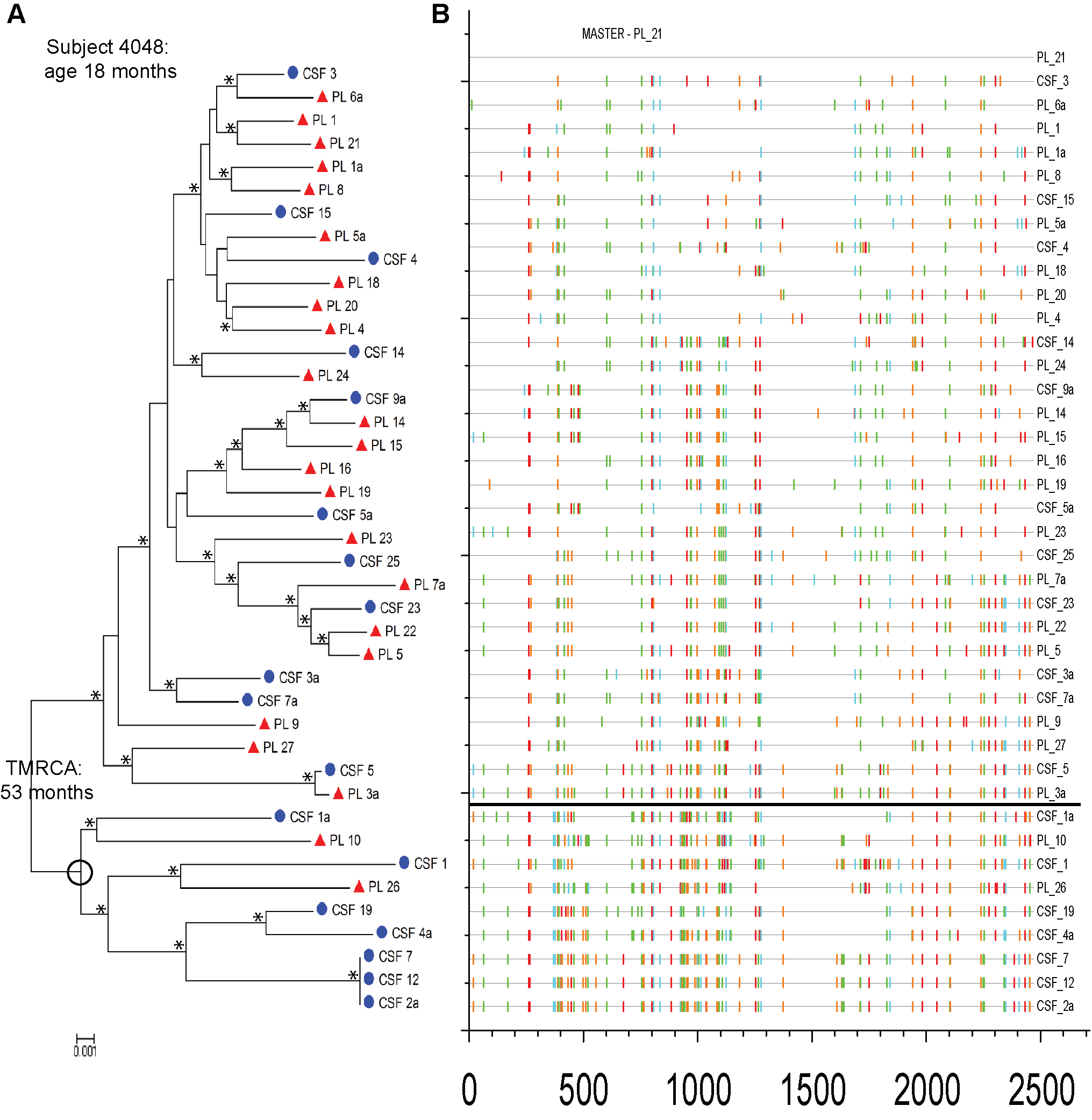

Supplement: Figure S4 — Intermediate subject 4048 exhibiting greater than two transmitted viruses. Phylogenetic and sequence analysis of plasma and CSF HIV-1 populations for subject 4048. (a) Neighbor-joining tree. Sequences from the CSF are labeled with solid blue circles, and plasma sequences (PL) are labeled with solid red triangles. Bootstrap values ≥40 are indicated (*) at the appropriate nodes. The phylogenetic tree characteristics are the same as those stated in Figure S3. (b) Highlighter plot of aligned env plasma and CSF sequences, generated at www.hiv.lanl.gov. The highlighter plot characteristics are the same as those stated in Figure S2. Several unique motifs were observed around 1000 base pairs (top), indicating that there were potentially greater than 2 transmitted viruses. Recombination between the transmitted viruses appears to account for much of the env genetic diversity detected in both the plasma and CSF populations. The transmitted CSF variant was maintained within the CNS, generating a minor CSF population with small local replication, accounting for the intermediate state observed for this subject. (TIF) [file ppat.1003094.s004.tif]

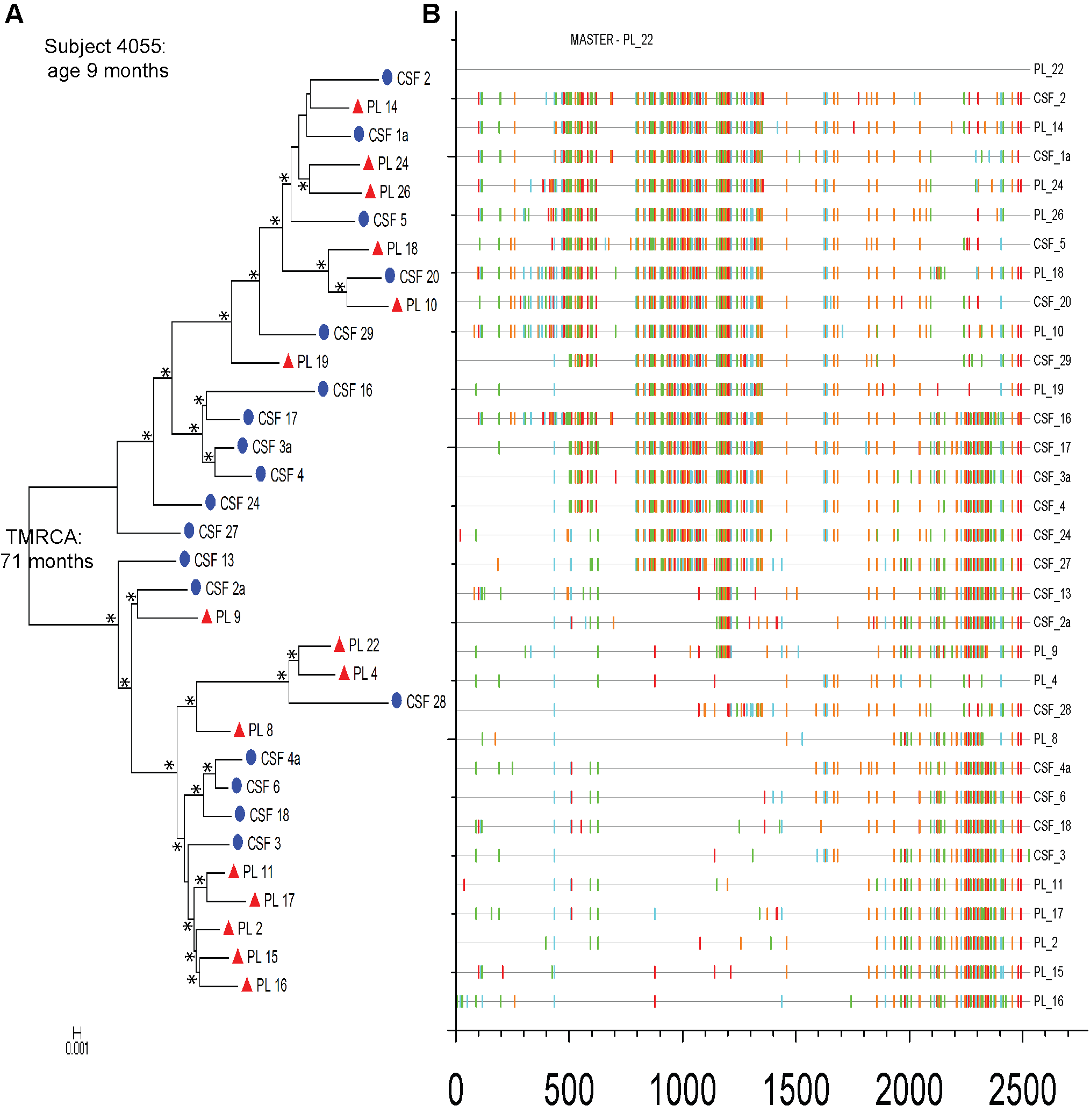

Supplement: Figure S5 — Equilibrated subject 4055 exhibiting two transmitted viruses. Phylogenetic and sequence analysis of plasma and CSF HIV-1 populations for subject 4055. (a) Neighbor-joining tree. Sequences from the CSF are labeled with solid blue circles, and plasma sequences (PL) are labeled with solid red triangles. Bootstrap values ≥40 are indicated (*) at the appropriate nodes. Genetic distance is scaled at the bottom of the figure (0.001) and indicates the number of nucleotide substitutions per site between env sequences. The subject's age is noted, as well as the overall TMRCA. (b) Highlighter plot of aligned env plasma and CSF sequences, generated at www.hiv.lanl.gov. The highlighter plot characteristics are the same as those stated in Figure S2. Subject was likely infected with 2 viral variants during transmission. The two sequences that are likely closet to the parental strains are CSF_16 and PL_4, and recombination between the transmitted viruses appears to account for much of the env genetic diversity detected in both the plasma and CSF populations. Both variants were maintained within the blood and CSF, accounting for the equilibrated state observed for this patient. (TIF) [file ppat.1003094.s005.tif]

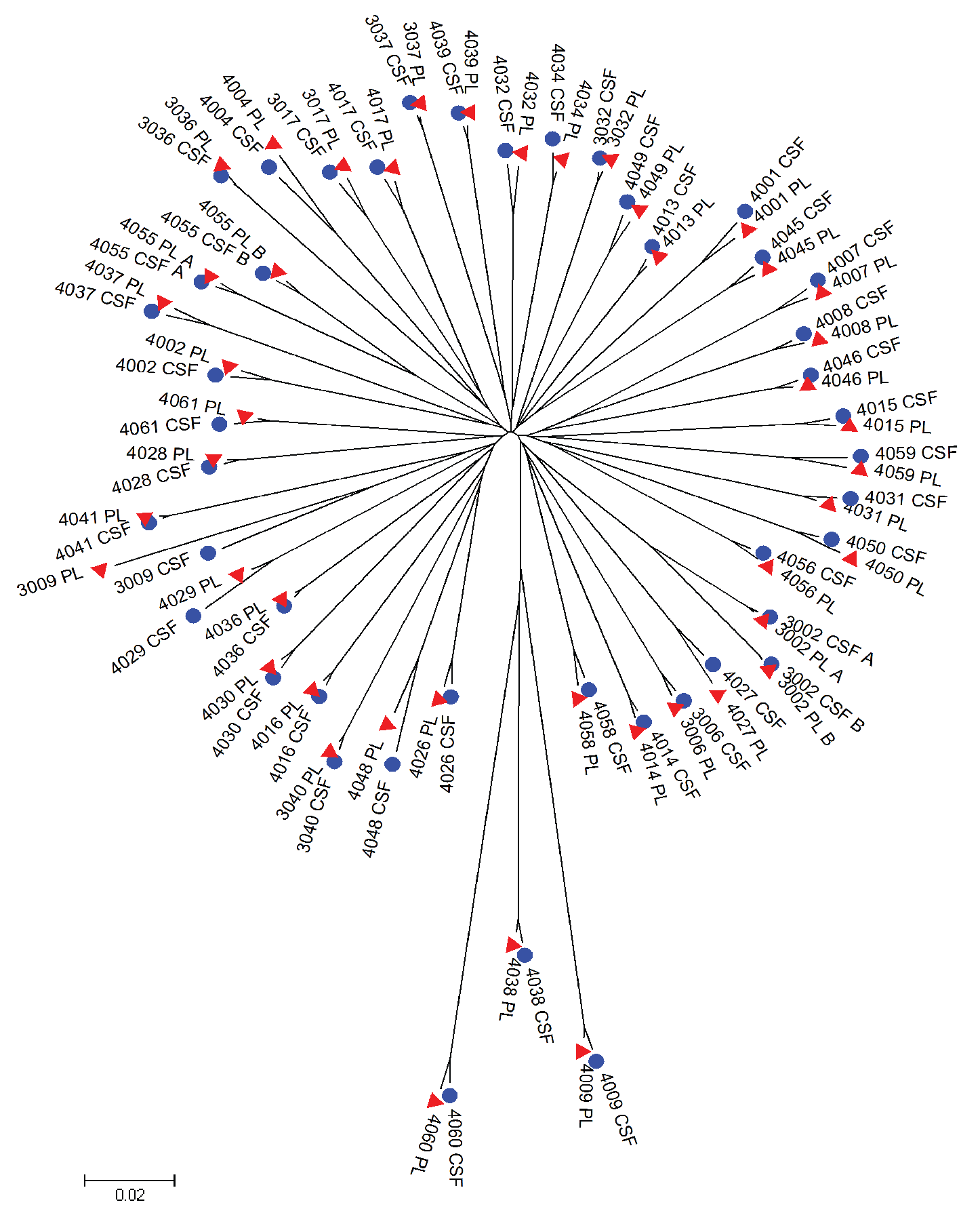

Supplement: Figure S6 — No contamination was observed between subjects. Neighbor-joining phylogenetic tree (radial topology). env sequences from the CSF are labeled with solid blue circles and env sequences from the blood plasma are labeled with solid red triangles. Genetic distance is indicated at the bottom of the figure and indicates the number of nucleotide substitutions per site between env sequences. Each subject ID is indicated. (TIF) [file ppat.1003094.s006.tif]
